# Isolated Bilateral Pedicle Fractures of L5 Without Underlying Predisposing Factors: A Rare Case Report

**DOI:** 10.3390/jcm14248719

**Published:** 2025-12-09

**Authors:** Jongyun Kwon, Seunghan Yu, Sang Hoon Jeong, Byung Chul Kim, Hyuk Jin Choi, Mahnjeong Ha

**Affiliations:** 1 Department of Neurosurgery and Medical Research Institute, Pusan Regional Trauma Center, Pusan National University Hospital, Busan 49241, Republic of Korea; kwonjongyun57@gmail.com (J.K.); inyourskull@naver.com (S.Y.); tkdgnsl5@naver.com (S.H.J.); bc1743kim@naver.com (B.C.K.); csfdiver@naver.com (H.J.C.); 2 School of Medicine, Pusan National University, Yangsan 50612, Republic of Korea

**Keywords:** isolated bilateral pedicle fracture, lumbar spine, low back pain, case report

## Abstract

We report a rare case of isolated bilateral pedicle fractures in the lumbar spine that occurred without any identifiable risk factors. Such fractures are uncommon, as they are typically accompanied by multiple other fractures. This type of fracture is commonly associated with widely acknowledged predisposing factors, including high-energy trauma, degenerative spine disease, previous spinal surgery, stress-related activities, or osteoporosis. Additionally, some reports suggest these fractures can result from low-energy trauma when underlying conditions such as osteoporosis are present. This report describes a 43-year-old female who presented with gradually aggravating low back pain in the absence of any significant trauma history. Initially, she denied any preceding injury, considering the event too trivial to mention. However, upon detailed history taking, she later recalled minor contact with the edge of her bed two days prior to symptom onset. Conservative management, consisting of administration of painkillers, adequate rest, the use of a brace, and rehabilitation exercises, led to significant improvement, with marked relief of clinical symptoms and fracture healing observed in follow-up imaging. Early identification and appropriate management of isolated pedicle fractures are essential, as delayed diagnosis may lead to chronic pain or long-term sequelae. Furthermore, unilateral fracture can increase mechanical loading on the contralateral pedicle, making it vulnerable to secondary stress injury. Therefore, clinicians must remain alert to the possibility of isolated pedicle fractures even in patients without risk factors. Thorough history taking is also essential, as unrecognized minor trauma may hinder timely diagnosis and optimal outcomes.

## 1. Introduction

Mechanical stress analyses have demonstrated that the pars interarticularis is less resistant to loading than the pedicle [1,2]. Consequently, isolated pedicle fractures in the lumbar spine are rarely observed. Isolated bilateral pedicle fractures are typically associated with high-energy trauma, degenerative spine diseases, previous spinal surgery, or predisposing factors [3,4,5,6,7]. Numerous studies have reported that, when caused by trauma, pedicle fractures are typically accompanied by other spinal fractures rather than occurring in isolation. According to previous reports, the most common symptom of an isolated pedicle fracture is low back pain (LBP) [8]. In some cases, radiating pain or motor weakness may occur, depending on the involvement of adjacent neural structures. Isolated pedicle fractures are often missed on plain radiographs because they are uncommon lesions and may not be clearly visualized due to overlapping anatomical structures and subtle fracture lines. Therefore, when persistent LBP occurs in patients with predisposing factors, an isolated pedicle fracture should be considered. In such cases, further diagnostic evaluation with computed tomography (CT) or magnetic resonance imaging (MRI) is recommended to ensure accurate detection and appropriate management [9,10,11]. However, as in this case, isolated pedicle fractures can also occur in the absence of any predisposing factors. In cases where the history of trauma is unclear or unrecognized, patients may fail to recall the event, emphasizing the importance of careful history taking to avoid diagnostic oversight. Management of isolated pedicle fractures should be determined based on the patient’s symptoms and spinal stability, with either conservative or surgical treatment options considered. Our case demonstrated complete clinical recovery, and follow-up imaging showed the healing process through conservative management. The authors report an uncommon case of isolated bilateral pedicle fractures and its successful treatment. This study was approved by the Institutional Review Board of our institute as a retrospective investigator-initiated trial (No. 2509-009-154). The Institutional Review Board waived the need for informed consent due to the retrospective nature of the study.

## 2. Case Descriptions

### 2.1. Presentation and Examination

A 43-year-old housewife began to experience LBP on 7 January 2024, without any history of significant trauma. According to the patient’s later report during hospitalization, the initial pain intensity at symptom onset was approximately 5 on the Numeric Rating Scale (NRS), and the discomfort was tolerable at that time. She had no prior history of degenerative spine disease and never experienced chronic LBP or any neurologic symptoms before this episode. Following symptom onset, her low back pain showed progressive worsening. She visited a local clinic two days later, on 9 January, where initial plain radiographs revealed no definite evidence of fracture. However, as her symptoms worsened, computed tomography was performed, which identified isolated bilateral pedicle fractures. Consequently, the patient was referred to our hospital on 17 January for further evaluation and management. The patient was admitted for further evaluation to identify any predisposing factors that might have led to the pedicle fractures. During hospitalization, a more detailed history was obtained. Upon additional questioning, the patient vaguely recalled having slightly bumped against the edge of her bed a few days before the onset of symptoms. The patient’s medical history included hypertension, which was controlled with medication, and previous surgery for hysterectomy due to uterine myoma and ovarian cyst removal. She had no predisposing factors such as degenerative spine disease, prior spinal surgery, high-energy trauma, or repetitive stress motion. Steroid use was carefully scrutinized for any prior exposure that could be associated with reduced bone density. However, no systemic or intermittent steroid use was identified. Additional confirmation obtained from the accompanying family member indicated no previously unreported medical conditions, prior surgeries, or medication use beyond what had already been obtained during the initial assessment. Dual-energy X-ray absorptiometry (DEXA) revealed normal bone mineral density in the lumbar spine, femoral neck, and total hip (L1–L4 T-score = 1.8; femoral neck T-score = 1.3; total hip T-score = 1.9), indicating that the patient did not have osteoporosis. At the time of visiting our clinic, the patient reported severe LBP with an NRS of 7, accompanied by gait disturbance. She was able to stand and walk without assistance, but the pain was aggravated by walking. On physical examination, localized tenderness was observed over the L5 region. No other neurological symptoms, including radiating pain or motor weakness, were observed.

**Figure 1:**
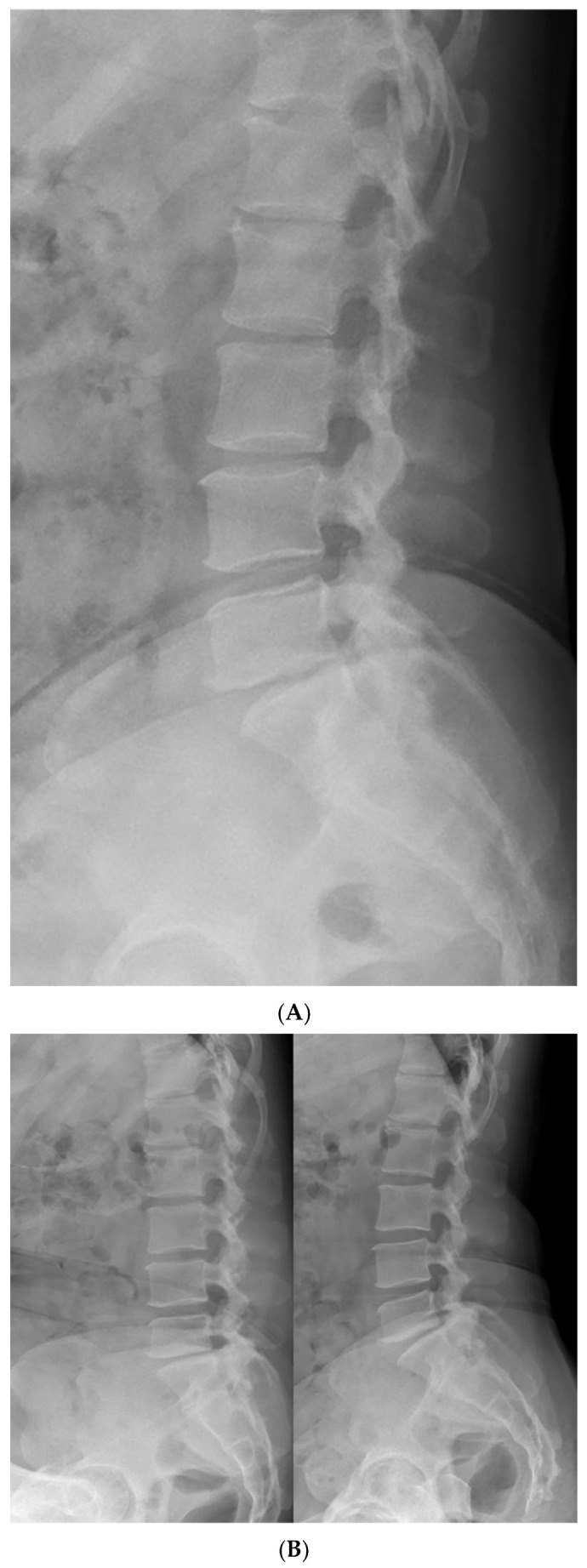

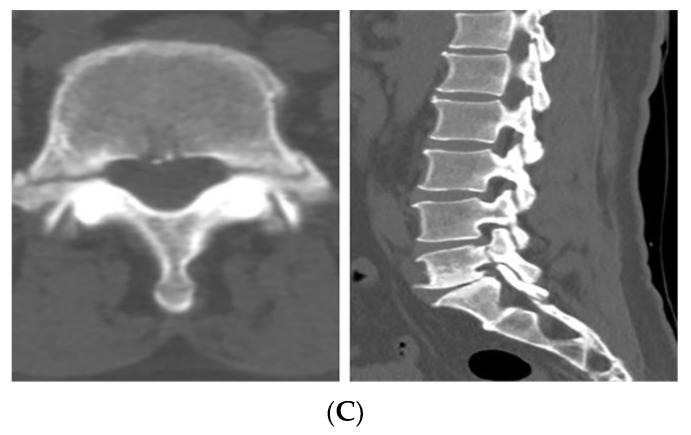
Initial radiologic evaluation at the first visit to our hospital. (**A**) The image shows a subtly visible fracture at the pedicle of L5 and Meyerding grade 1 spondylolisthesis at the L5–S1 level. (**B**) Flexion–extension lateral radiographs showing no relative sagittal plane translation between L5 and S1. (**C**) CT images confirming isolated bilateral pedicle fractures at L5 without additional fractures. No sclerotic change, pseudoarthrosis, or pars defect was observed, consistent with an acute fracture.

**Figure 2:**
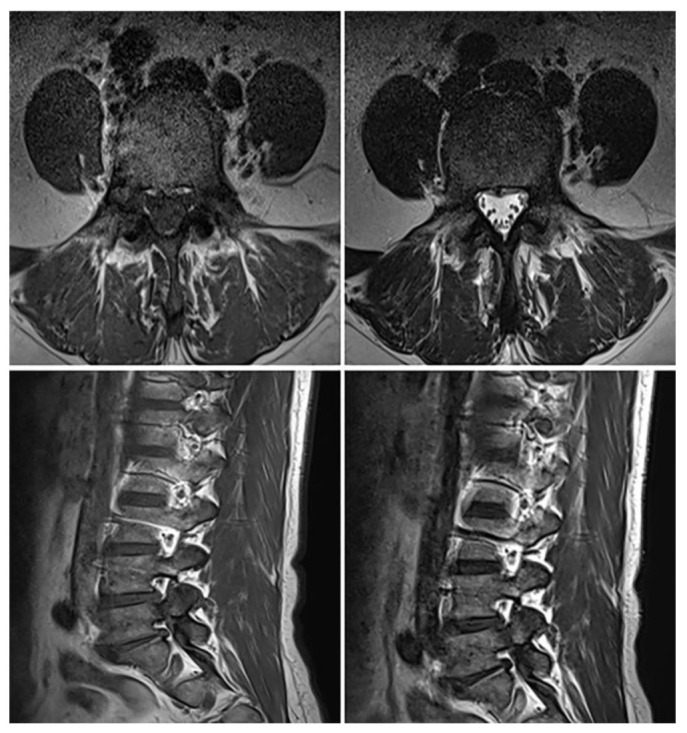
MRI showing low signal intensity on T1- and T2-weighted images in the bilateral L5 pedicles with minimal hyperintense marrow edema on T2, consistent with acute fractures. Additional findings include central disc protrusion at L3–4 and L4–5, and disc bulging with Modic type 2 change at L5–S1.

Further imaging evaluation was performed to confirm the previously diagnosed L5 bilateral pedicle fracture and to assess the involvement of surrounding structures. Figure 1A shows a plain radiograph taken during the initial examination in our hospital, revealing a subtly visible fracture at the pedicle of L5 and suspicious Meyerding grade 1 spondylolisthesis at the L5-S1 vertebrae. As shown in Figure 1B, dynamic flexion-extension lateral radiographs were performed to assess instability that could affect the treatment approach, and no abnormal findings were observed. Figure 1C shows a CT scan confirming isolated bilateral pedicle fractures at L5, consistent with the findings from the previous examination at the local clinic, and no additional fractures were identified. There were no sclerotic changes surrounding the fracture lines or pseudoarthrosis, suggesting an acute fracture despite the absence of recent trauma history. Pars defects or hypoplastic neural arch were not observed. Figure 2 shows MRI findings demonstrating low signal intensity on T1 and T2 weighted images in the bilateral pedicle lesions, consistent with acute lesions, and minimal hyperintense marrow edema surrounding the fracture lines on T2. Additionally, imaging revealed mild degenerative changes, including disc bulging at L3–4, L4–5, and L5–S1, and Modic type 2 at L5–S1. These disc lesions showed no features suggestive of an acute rupture and were more compatible with chronic degenerative changes rather than acute pathology. Given the absence of pre-existing LBP or neurologic symptoms, these degenerative findings were regarded as incidental and unrelated to the patient’s current presentation.

To determine the appropriate treatment plan, the imaging findings were reviewed together with the patient’s clinical presentation. CT scan demonstrated clearly defined fracture lines in the bilateral L5 pedicles without any sclerotic change, along with a sharp, non-corticated fracture margin. MRI showed a linear low-signal fracture line on both T1- and T2-weighted sequences without evidence of bone marrow remodeling. These features indicated that the lesion represented an acute fracture that required treatment. Treatment options for pedicle fractures can be divided into surgical and conservative treatments. However, isolated bilateral pedicle fractures are rare, and no standardized treatment guidelines have been established. Given the pedicle’s essential role in spinal stability, screw fixation may be considered, but previous reports indicate that surgery has primarily been performed when thecal sac compression, foraminal stenosis with neurologic symptoms, or mechanical instability are present. In the present case, flexion–extension radiographs revealed no segmental slippage or abnormal angulation indicative of instability. No thecal sac compression was observed, and although mild foraminal stenosis from underlying degenerative changes was present, there was no indentation of the exiting L5 nerve root and no radicular symptoms. Moreover, El Rachkidi et al. and Surur et al. reported that patients with L5 pedicle fractures without instability or neurologic symptoms achieved complete pain relief and radiographic consolidation with conservative treatment, resulting in patients showing high satisfaction [9,12]. Furthermore, reports describing surgical management also emphasize that operative treatment was typically reserved for cases in which conservative therapy had failed, significant neurologic symptoms developed, or progressive mechanical instability was identified. In patients presenting predominantly with localized low back pain without neurologic deficits, primary screw fixation was rarely performed, and surgery was considered only when pain worsened despite an adequate conservative management. In addition, when symptoms are not severe the potential for long-term complications associated with pedicle screw fixation including adjacent segment degeneration must be taken into consideration before surgical intervention [13]. Finally, the patient expressed a clear preference for non-operative management. Based on these findings, sufficient pain relief and functional recovery were expected with conservative management, as supported by previous literature. Therefore, conservative treatment was initiated.

### 2.2. Post-Treatment Course

**Figure 3:**
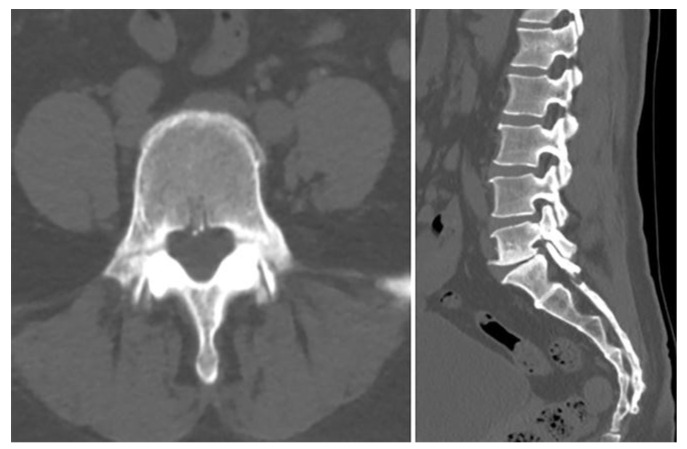
Follow-up CT images showing sclerotic changes around the fracture site with partial bone bridge formation, indicating progressive healing.

Following the decision to proceed with conservative management, the patient was prescribed acetaminophen, tramadol, and eperisone for pain control, and a thoracolumbosacral orthosis (TLSO) was applied. Light ambulation was permitted, while activities that could strain the lower lumbar regions such as twisting the torso or sitting on the floor were restricted. During the 9-day hospital stay, the patient’s LBP improved from an NRS of 7 at admission to 3, and she gradually experienced less pain during ambulation, leading to her discharge with follow-up in the outpatient clinic. 27 days after discharge, the patient visited the outpatient clinic with minimal pain and no neurological symptoms. Follow-up lumbar spine radiographs (anteroposterior, lateral, flexion, and extension views) revealed no evidence of instability or additional fracture. On the 69th day after discharge, the patient visited to the outpatient clinic pain-free and no longer required medication. CT demonstrated sclerotic changes surrounding the fracture site with partial bone bridge formation, as shown in Figure 3.

**Figure 4:**
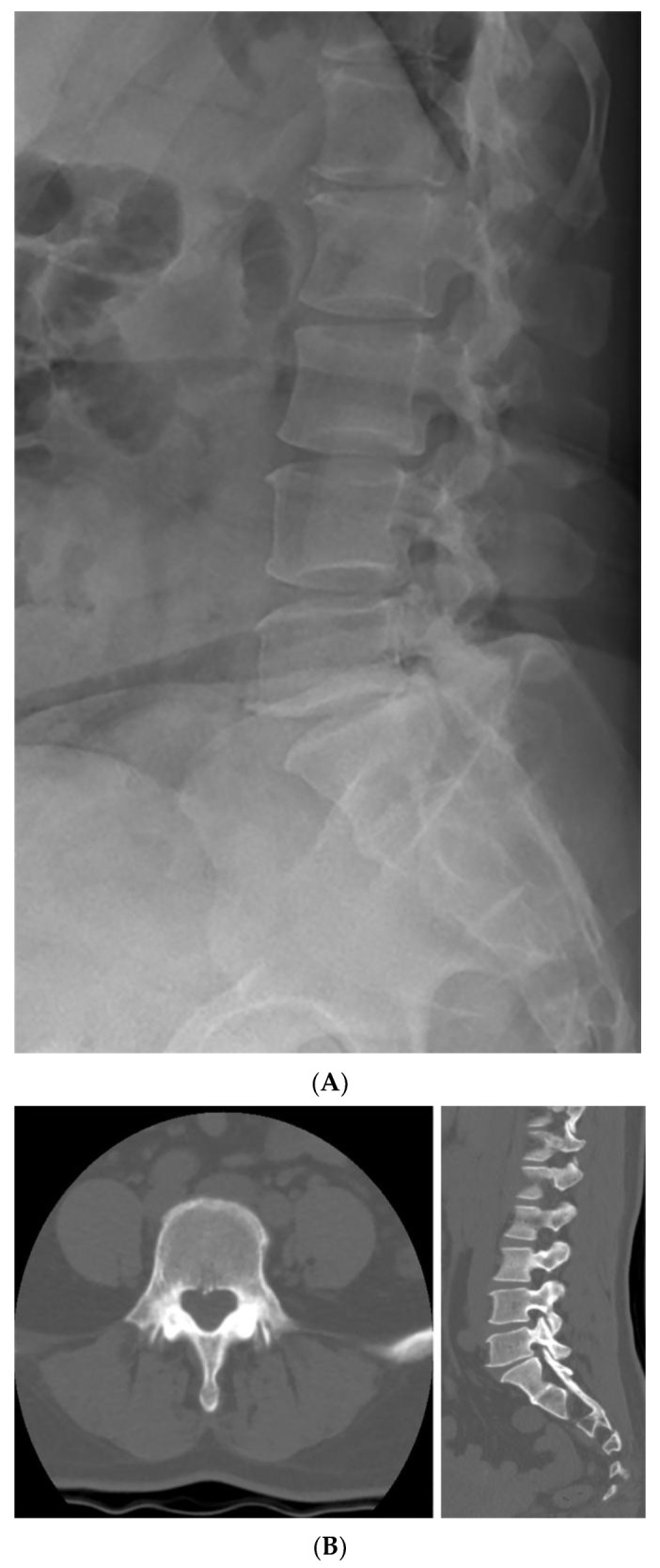
Four-month follow-up plain radiograph and CT scan after the injury. (**A**) The plain radiograph suggests almost complete bony union of the bilateral L5 pedicle fractures, although clear assessment of the fracture lines is limited. (**B**) The CT scan shows sclerotic change around the fracture margins and an interval reduction in the fracture lines.

Although the patient’s symptoms had improved, imaging findings suggested that further bone healing was required. Therefore, the duration of brace application was extended by one additional month beyond the initially planned three months. At the four-month follow-up visit after the injury, a plain radiograph, dynamic flexion–extension lateral radiographs, and a CT scan were conducted. On plain radiographs, the bilateral L5 pedicle fractures appeared to show almost complete bony union, although the fracture lines could not be clearly assessed. Dynamic flexion–extension radiographs demonstrated no evidence of instability. To further determine whether additional bracing or activity restrictions were required, a CT scan was obtained for more detailed evaluation. The CT images allowed a more precise assessment and demonstrated cortical thickening with sclerotic change around the fracture margins, bone bridge formation, and fracture gap narrowing, indicating interval reduction in the fracture lines and near-complete union. Pedicle stress reaction was also assessed on follow-up imaging. Stress-related changes such as diffuse pedicle sclerosis, excessive cortical hypertrophy, absence of fracture gap narrowing, and the presence of new fissures or lucencies were specifically checked. None of these findings were observed, indicating no evidence of persistent pedicle stress. (Figure 4). The patient was permitted to remove the brace and resume normal activities without motion restrictions. The patient expressed satisfaction that surgical treatment was not required, although wearing the brace for a long period was uncomfortable. However, she stated that the brace greatly helped relieve her pain. The total follow-up duration was four months from the onset of injury. The patient’s symptoms progressed from an NRS score of 5 to 7 on admission, but improved to an NRS score of 3 with conservative management, including medication, TLSO application, and activity restriction during hospitalization. By the four-month follow-up, her pain had completely resolved, and imaging studies demonstrated near-complete bony reunion of the bilateral pedicle fractures.

## 3. Discussion

As noted above, isolated bilateral pedicle fractures are rarely observed in clinical practice, especially when high-energy trauma, previous spinal surgery, or other known risk factors are absent [14]. This can be attributed to the fact that pedicle fractures are often accompanied by injuries to the vertebral body or other posterior elements [15], and that fractures more commonly occur at the pars interarticularis due to its relatively weaker mechanical strength compared to the pedicle [16,17]. Multiple biomechanical mechanisms have been suggested to account for pedicle fractures, particularly when no obvious triggering conditions are evident. When exposed to altered loading dynamics, such as segmental instability, lever-arm effects or localized stress at the transverse process, the pedicle may become vulnerable to injury, particularly from repeated shear and torsional forces. Additionally, during certain movements involving repeated axial loading or twisting forces, the transverse process can act like a lever that transfers excessive mechanical force to the pedicle [18]. Over time, this repeated stress can cause a fatigue-type fracture, even if the vertebral body or pars interarticularis itself remains intact.

Currently, because isolated pedicle fractures are extremely rare, there is no clear consensus regarding their optimal management. Clinical decisions must therefore be individualized, with consideration of factors including spinal instability, neurologic symptoms, and the patient’s activity level. According to previous studies, conservative treatment consisting of rest, activity modification, bracing, and physical therapy has been reported as effective in the absence of spinal instability or neurological deficit [8,9,10].

On the other hand, surgery should be considered when conservative treatment fails, including cases of worsening low back pain, new neurological deficits, or the development of instability [19,20,21]. Transforaminal lumbar interbody fusion (TLIF) with transpedicular screw fixation is often considered the standard surgical option for pedicle fractures occurring adjacent to instrumented segments or de novo pedicle stress fractures. However, fusion procedures may lead to long-term complications, including adjacent segment degeneration. To avoid the adverse effects associated with segmental fusion, simple fixation using traction screws has been introduced as an alternative technique [13]. In the case reported by Kögl et al., the patient presented with acute low back pain (NRS 7) accompanied by L5 radicular pain. Conservative treatment was first attempted, but the symptoms worsened. Accordingly, subsequent evaluation was performed, which revealed possible compression of the L5 nerve root caused by mobile pedicle fracture fragments on flexion–extension radiographs. Because the fracture fragments resulted in radiculopathy, minimally invasive reduction and fixation with traction screws were performed. Notably, even in this report, conservative management was the initial treatment, and surgery was reserved until symptoms worsened due to nerve root indentation. Although traction screw fixation is a less invasive and promising alternative to fusion, it is still associated with potential procedure-related complications. In patients with diminished bone quality, complications related to screw fixation are more likely to occur. Additionally, altered loading dynamics following instrumentation can increase adjacent segment stress. Therefore, when low back pain is the primary symptom and no neurological deficit is present, conservative treatment should be considered first, as symptom resolution and complete bony union can be expected.

Although only a few cases of isolated pedicle fractures have been reported in the literature, the current case is distinct in several aspects. Unlike previously reported cases, this case presented with bilateral rather than unilateral pedicle fractures, and the patient had no identifiable risk factors such as osteoporosis, repetitive mechanical stress, or prior spinal surgery. Moreover, the clinical presentation was limited to progressive low back pain, and initial plain radiographs failed to reveal any definitive lesions. Given that isolated bilateral pedicle fractures are uncommon and may present with nonspecific symptoms, there is a significant risk of misdiagnosis, particularly in primary care settings where further evaluation with CT or MRI can be limited. In such contexts, clinicians may overlook the possibility of a pedicle fracture, especially in the absence of high-energy trauma or known predisposing factors. However, as demonstrated by the present case, isolated pedicle fractures can occur even without these typical risk factors. Moreover, as in the present case, it is possible that patients may initially deny trauma when the event is trivial and not perceived as an injury. Therefore, when persistent LBP is present and the underlying cause remains unclear, maintaining clinical suspicion and obtaining a detailed history to identify possible minor trauma may facilitate earlier diagnosis and prevent delayed management, even when advanced imaging is not available. Furthermore, in patients with persistent or progressive pain, careful physical examination to evaluate localized tenderness at the affected area is essential. Under these circumstances, when a detailed history reveals even minor trauma and the patient presents with persistent or progressive low back pain accompanied by localized tenderness on physical examination, further evaluations such as CT or MRI should be considered. In addition, the presence of any neurologic symptoms, including radiating pain, clearly requires further imaging work-up. However, as a single case report, the findings cannot be generalized. The absence of long-term follow-up and biomechanical testing limits the ability to fully elucidate the underlying causative factors and long-term prognosis.

## 4. Conclusions

This case highlights several important clinical considerations regarding isolated pedicle fractures. First, persistent or progressively worsening LBP should not be underestimated, even when there is no apparent history of significant trauma. A thorough physical examination should always be performed to assess localized tenderness. Second, although rare, isolated pedicle fractures can occur without specific predisposing factors. Careful evaluation of plain radiographs is essential, and when a fracture is suspected, further imaging with CT or MRI should be performed to avoid misdiagnosis or delayed detection. Finally, treatment decisions should be guided by two major principles: the stability of the spinal structure and the presence or absence of neurological symptoms. Conservative management can be effective when spinal stability is maintained and neurological deficits are absent, whereas surgical intervention should be reserved for cases with instability or persistent neurological compromise. In summary, maintaining clinical suspicion in cases of unexplained or persistent low back pain, combined with appropriate imaging and individualized treatment planning, is key to ensuring timely diagnosis and optimal outcomes.

## Data Availability

The data presented in this study are available on request from the corresponding author.
